# Analyzing Optimal Wearable Motion Sensor Placement for Accurate Classification of Fall Directions

**DOI:** 10.3390/s24196432

**Published:** 2024-10-04

**Authors:** Sokea Teng, Jung-Yeon Kim, Seob Jeon, Hyo-Wook Gil, Jiwon Lyu, Euy Hyun Chung, Kwang Seock Kim, Yunyoung Nam

**Affiliations:** 1 Department of ICT Convergence, Soonchunhyang University, Asan 31538, Republic of Korea; teng.sokea@sch.ac.kr; 2 ICT Convergence Research Center, Soonchunhyang University, Asan 31538, Republic of Korea; betterwithme@sch.ac.kr; 3 Department of Obstetrics and Gynecology, Soonchunhyang University Cheonan Hospital, Cheonan 31151, Republic of Korea; sjeon4595@gmail.com; 4 Department of Internal Medicine, Soonchunhyang University Cheonan Hospital, Cheonan 31151, Republic of Korea; hwgil@schmc.ac.kr; 5 Division of Respiratory Medicine, Department of Internal Medicine, Soonchunhyang University Cheonan Hospital, Cheonan 31151, Republic of Korea; 78214@schmc.ac.kr; 6 Department of Dermatology, College of Medicine, Soonchunhyang University Cheonan Hospital, Cheonan 31151, Republic of Korea; djavan@naver.com; 7 Future Innovation Medical Research Center, Soonchunhyang University Cheonan Hospital, Cheonan 31151, Republic of Korea; kimks5005gt@gmail.com; 8 Department of Computer Science and Engineering, Soonchunhyang University, Asan 31538, Republic of Korea

**Keywords:** falls, optimal sensor, fall direction, classification, machine learning, feature extraction, IMU sensors, daily life activity, optimal sensor, sensor location

## Abstract

Falls represent a significant risk factor, necessitating accurate classification methods. This study aims to identify the optimal placement of wearable sensors—specifically accelerometers, gyroscopes, and magnetometers—for effective fall-direction classification. Although previous research identified optimal sensor locations for distinguishing falls from non-falls, limited attention has been given to the classification of fall direction across different body regions. This study assesses inertial measurement unit (IMU) sensors placed at 12 distinct body locations to determine the most effective positions for capturing fall-related data. The research was conducted in three phases: first, comparing classifiers across all sensor locations to identify the most effective; second, evaluating performance differences between sensors placed on the left and right sides of the body; and third, exploring the efficacy of combining sensors from the upper and lower body regions. Statistical analyses of the results for the most effective classifier model demonstrate that the support vector machine (SVM) is more effective than other classifiers across all sensor locations, with statistically significant differences in performance. At the same time, the comparison between the left and right sensor locations shows no significant performance differences within the same anatomical areas. Regarding optimal sensor placement, the findings indicate that sensors positioned on the pelvis and upper legs in the lower body, as well as on the shoulder and head in the upper body, were the most effective results for accurate fall-direction classification. The study concludes that the optimal sensor configuration for fall-direction classification involves strategically combining sensors placed on the pelvis, upper legs, and lower legs.

## 1. Introduction

Falls are a major public health concern, ranking as the second leading cause of injuries and accidental death globally. They can lead to severe physical and emotional repercussions, including impairment, loss of autonomy, post-fall syndrome, depression, reduced activity levels, and possibly early mortality [1]. Wearable sensor systems significantly enhance the well-being of patients and older adults by assessing fall risk, detecting falls, and monitoring other health-related factors [2]. These systems typically place sensors on body areas like the waist, chest, and thighs to track daily activities, estimate energy expenditure, aid rehabilitation, and monitor heart activity [3,4].

The accuracy and efficiency of classification or detection systems are significantly influenced by sensor placement on the body. Previous studies have primarily focused on identifying optimal sensor locations for distinguishing between fall events and non-fall activities or activities of daily living (ADLs). Our study builds upon this by specifically classifying fall directions, including forward fall, backward fall, and sideways fall, in addition to non-fall activities such as sitting, standing, walking, and transitions between sitting and standing. While the waist is often recommended as an optimal location for fall detection due to its proximity to the body’s center of mass, placement on the arms and legs is generally discouraged. This is because higher acceleration in these areas can impair the performance of detection systems, as noted in previous research [5].

Optimal performance varies by location, with several studies suggesting the waist, chest, thigh, or right pocket as effective sites for fall detection and classification [6,7,8,9,10,11]. Notably, the waist and thigh are often highlighted for their ability to capture comprehensive movement dynamics essential for accurate fall detection and classification [12,13]. In previous studies, fall detection focused on identifying whether a fall event occurred. It is important for systems that trigger alerts or further monitoring. However, the purpose of fall classification is to categorize the type or direction of the fall, such as forward, backward, or sideways. Classifying the direction of a fall is crucial because different injuries occur depending on the direction of impact, and this information is critical for tailoring medical interventions. For example, forward falls may result in wrist or arm fractures, while backward falls are more likely to cause head or hip injuries. By classifying fall direction, we can better understand the biomechanics of the fall and design more effective interventions.

Studies have shown that sensor location profoundly influences fall detection and classification accuracy. Identifying optimal body locations for IMUs is essential, particularly because different age groups prefer using the minimum number of wearable devices [14,15,16]. Common sensor locations for fall classification include the waist, chest, and thigh, with alternatives such as the forehead, neck, ear, shoulder, back, wrist, ankle, and foot, though these locations are generally less favorable [17,18,19,20,21,22,23,24,25]. For instance, Ponce et al. [5] reinforced the significant impact of sensor placement by determining that a combination of sensors at the waist and a lateral viewpoint provided the best results for fall detection, while Martínez-Villasenor et al. [6] found that waist sensors achieved 95.76% accuracy in fall classification using the UP-Fall dataset with various machine learning techniques, including random forest (RF), support vector machine (SVM), and multilayer perceptron (MLP). Santoyo et al. [7] found that sensors on the chest, waist, or both provided the best results using KNN, SVM, NB, and DT classifiers. Moreover, Özdemir et al. [12] demonstrated that a single waist sensor provided the best performance when analyzing 31 sensor combinations. Ntanasis et al. [13] highlighted that sensors placed on the waist and thigh were most effective for fall detection and daily activities monitoring. Table 1 summarizes key studies comparing sensor placements for optimal fall detection.

While several studies focus on fall detection, some studies specifically address fall-detection classification, a key method for determining fall direction. Accurate fall-direction classification requires comprehensive data, making it essential to identify the body regions that provide the most relevant information. For example, the authors in [26] used IMUs and eight machine learning classifiers with accelerometers, gyroscopes, and magnetometers placed on the waist for fall-direction classification. Despite the substantial body of research on fall detection, the optimal sensor locations for this purpose remain undetermined.

Proper sensor placement is crucial for accurately capturing falls and activities of daily living (ADLs), especially for distinguishing fall directions. Therefore, our study divided the body into upper and lower regions to identify the most effective sensor combination locations for fall-direction classification, which is crucial for understanding the biomechanics of falls and for implementing effective fall prevention strategies. Distinguishing the direction of a fall holds significant clinical and scientific importance, as the direction can directly affect the likelihood and severity of injuries, such as fractures or head trauma. For instance, forward falls often lead to wrist or arm fractures as individuals attempt to break their fall, whereas backward falls are more likely to result in head or hip injuries.

Classifying the direction of a fall is essential because different injuries occur depending on the direction of impact, and this information is critical for tailoring medical interventions. Clinicians often require detailed fall-direction data to guide treatment decisions, especially when the patient has lost consciousness and cannot describe how they fell. Knowing the fall direction can help medical professionals identify which body parts may have been impacted and adjust treatment accordingly. For example, a backward fall might pay more attention to the head and hips, whereas a forward fall might require focusing on the arms or wrists. Robinovitch et al. [27] have demonstrated that injury risk and protective responses vary significantly depending on fall direction. Thus, accurately identifying fall direction is key to developing personalized interventions and preventive measures, particularly for vulnerable populations such as older adults who are at higher risk of falls and injuries. This understanding can also inform the development of targeted fall prevention technologies and clinical interventions to reduce injury risks in high-risk populations.

This approach is driven by the distinct movement dynamics of the upper and lower body during fall events, which may influence the performance of fall-direction classification systems. For example, sensors on the lower body, such as the ankle or thigh, capture different fall aspects compared to sensors on the upper body, such as the chest or head. No more research has systematically explored sensor placement by dividing the body into upper and lower regions, leaving a gap in understanding that locations provide the most relevant data. This division also addresses the need for flexibility in sensor placement owing to discomfort, medical reasons, or physical limitations, catering to specific populations like the elderly or athletes. For instance, elderly individuals with lower-body weakness may benefit from lower-body sensors, while athletes might require upper-body sensors. This separation facilitates flexible sensor placement tailored to the specific needs of each target population.

This research addresses the gap by investigating the impact of sensor placements on the efficiency of fall-direction classification. We analyzed multiple body locations to identify the most effective sensor positions and employed advanced machine learning techniques to evaluate classification accuracy across various regions of the body.

The primary aim of this study was to determine the optimal sensor placements among 12 body locations and ascertain the critical body positions for recognizing and classifying fall directions using wearable IMU sensors. The key contributions of this study are as follows:

- Identify the most effective machine learning classifier for fall-direction analysis using data from 12 body locations;
- Examine how the left and right body sides affect fall-direction classification accuracy;
- Determine the most effective sensor location combinations for upper and lower body parts;
- Utilize a multiclass classification framework to evaluate and compare the effectiveness of sensor placement in identifying specific fall directions (e.g., forward, backward, and lateral) and non-falls.

The remainder of this paper is organized as follows: Section 2 explains the dataset and methodology for comparative analysis, Section 3 presents the results and analysis, Section 4 presents a discussion of results, and the conclusion summarizes the study.

**Table 1:** Summarizing relevant work on finding the best sensor placements for fall detection.

Ref	Dataset	Sensor	Sensor Location	Subject	Combination	Falls, ADLs	Algorithm	Best Performance
[5]	UP-Fall	5 IMUs, 1 helmet, 6 ambient, and 2 cameras	Neck, waist, left wrist, right pocket’s trousers, and left ankle	17	5 IMU, 2 cameras	5/6	RF, SVM, MLP, KNN	- Waist and a lateral viewpoint - Acc: 98.72% (RF) - Right pocket acc: 98.57% (RF) - Wrist, acc: 98.32% (RF)
[6]	UP-Fall	5 IMUs, 1 helmet, 6 ambient, and 2 cameras	Neck, waist, left wrist, right pocket’s trousers, and left ankle	17	Single	5/6	RF, SVM, MLP, KNN	Waist (RF) acc: 95.76%
[7]	UMA-Fall	1 smartphone and 4 IMU	Thigh pocket, chest, waist, right wrist, and ankle	19	31	3/11	SVM, KNN, NB, DT	Waist, chest: sen: 95% using SVM
[12]	Daily and Sports Activities	6MTw sensor (six three-DOF)	Right wrist, right thigh, right ankle, chest, waist, and head	14	63	20/16	KNN, BDM, SVM, LSM, DTW, ANNs	Waist: acc 99.87% using k-NN
[13]	Daily and Sports Activities	Acc + Gyr + Mag	Heads, chests, waists, right wrists, right thighs, and right ankles	14	Single	20/16	WEKA J48, DT, KNN, RF, RC, SVM	Waist: acc 99.28% using RF Thigh: acc 99.48% using SVM

## 2. Methodology

This section outlines the overall procedure for identifying the best sensor locations to classify fall directions. First, we collected IMU data from 12 sensor placements on the human body. The proposed methodology involves defining scenarios for selecting specific sensor locations and segmenting body parts. Then, we developed supervised learning models to assess these locations. Statistical analyses were employed to determine the most effective sensor placements for each body part and to compare different body regions for optimal and accurate fall-direction classification.

### 2.1. Acquisition

The study included 24 participants (13 females and 11 males) with ages ranging from 18 to 50 years and heights from 155 cm to 183 cm. The dataset was collected from various environments, including hospitals, homes, roads, and nursing homes. The inclusion criteria required participants to be physically healthy, with no musculoskeletal problems or neurological conditions problem that could impair their ability to perform falls. Participants with any injuries or mobility issues were excluded from the study to ensure safety during fall simulations. While participants were not at high risk of falls, they were trained to simulate realistic fall behaviors based on knowledge of fall risk dynamics. This allowed the participants to imitate falls typically experienced by individuals at a higher risk of falling, ensuring realistic and accurate fall simulations. 

The data collection involved the use of three types of IMU sensors—a 3D accelerometer, 3D gyroscope, and 3D magnetometer—attached to 12 body locations, including the head, shoulders, upper arms, forearms, pelvis, upper legs, and lower legs. These sensors recorded movement data across three axes (X, Y, and Z). Participants were instructed to self-initiate falls in three different directions: backward, forward, and lateral (side falls). Non-fall activities, such as sitting, standing, walking, and transitioning between sitting and standing, were also recorded to provide control data for distinguishing between fall and non-fall events. Specifically, participants were asked to fall 1–10 times in each direction and each place depending on the participant’s ability and comfort level. These activities were chosen to represent common daily movements that could potentially be misclassified as falls. The dataset was classified into four categories: non-fall, backward fall, forward fall, and lateral (side) fall. Table 2 provides a detailed breakdown of the sample counts for each category and the maximum row count per sample, ensuring a robust dataset for fall detection and classification analysis.

### 2.2. Body Parts Division and Sensor Selection from Each Part

**Figure 1:**
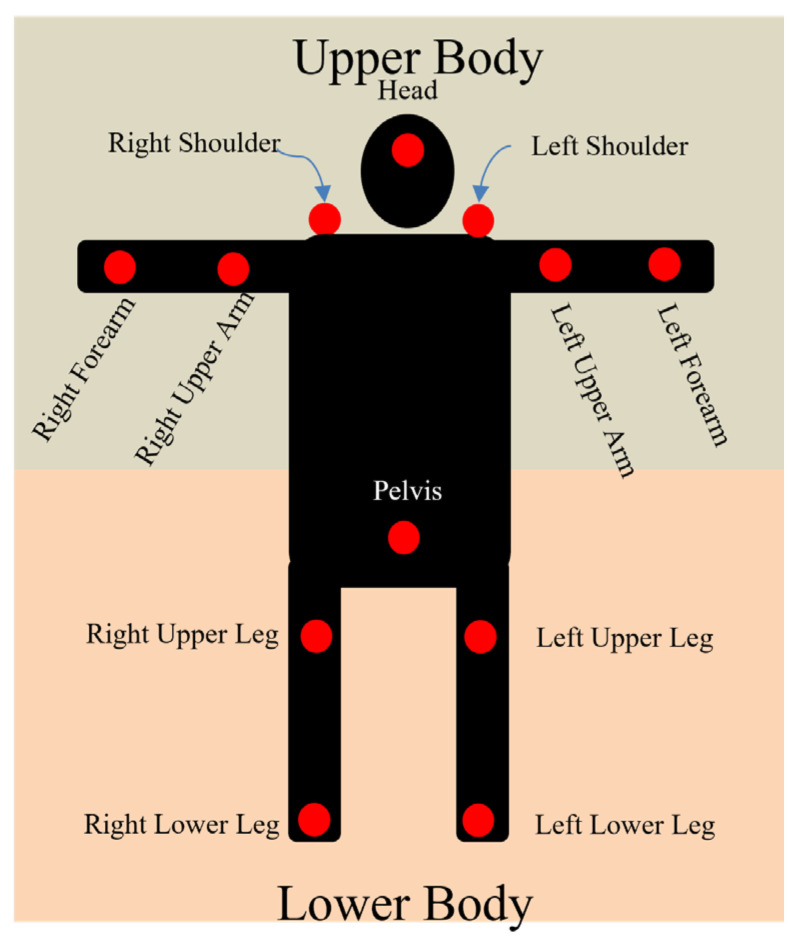
Description of wearable sensor placements and body region divisions.

To identify the most effective sensor locations for fall classification, we divided the body into two main regions: upper and lower. This division was based on [28], which categorized the body into three parts and later into more than three parts. In our study, the upper body includes the head, shoulders, upper arms, and forearms, whereas the lower body consists of the pelvis, upper legs, and lower legs. This division is illustrated in Figure 1. 

The rationale behind dividing the body into upper and lower regions is to evaluate how each area contributes to fall-direction classification. This approach allows us to identify which sensors provide the most relevant information from each body region, enabling the selection of effective sensor configurations that minimize redundancy. By comparing the performance of upper- and lower-body sensors separately, we aimed to determine the optimal sensor locations for accurately capturing fall dynamics while also considering user comfort and practical placement considerations.

For each body part, we employed a factorial design approach, *N* = 2*^k^* − 1, where *k* represents the number of selected sensor positions [7], to study the effect of sensor placement on detection accuracy. *N* denotes the total number of possible combinations for each body region. For the upper body, with four selected sensors, there are 2^4^ − 1 = 15 possible combinations, and for the lower body, with three sensors, there are 2^3^ − 1 = 7 combinations (for the reasons behind selecting the position of each one, see Section 3.2). This approach allowed us to systematically explore different sensor placements to capture fall-direction information and identify the optimal configuration to enhance fall-direction classification performance. 

### 2.3. Materials and Methodologies

This section details the materials and methodologies used to identify the most effective sensor locations for fall classification using 12 wearable IMU sensors (Figure 1). Our analysis involved three main steps: (i) preprocessing and labeling, (ii) feature extraction, and (iii) building models to identify the most effective classifier, analyze differences between body sides, evaluate sensor combinations across upper and lower body regions, and validate the statistical significance of the results.

#### 2.3.1. Preprocessing and Labeling

To improve model performance and reduce noise, we applied a 1D Gaussian filter during data processing and labeled the data for classification. Each dataset file contained up to 600 samples recorded at 60 Hz, representing 10 s of data per sequence. The data were classified into four categories for supervised learning: non-falls, backward falls, forward falls, and lateral falls. The IMU sensor data were synchronized with video recordings of each fall event and labeled by human experts on fall events. Trained observers reviewed the video footage to accurately classify the fall direction (e.g., forward, backward, or lateral). This method ensured objective and consistent labeling, minimizing potential bias that could arise from self-reporting. The labeled data were subsequently used to train and evaluate machine learning models for fall-direction classification. 

Signal vector magnitude (*M*) was used to simplify the accelerometer, gyroscope, and magnetometer data by reducing the three-dimensional vectors (*x*, *y*, and *z*) into single scalar values, making pattern analysis and event detection more efficient [29]. This approach was chosen to minimize noise from individual axis variations, ensuring a more consistent representation of movement dynamics across different fall events. While analyzing individual axes (*x*, *y*, and *z*) could offer more detailed data for fall-direction classification, using *M* provided a more robust and simplified method for detecting fall-related patterns. In our study, *M* was integrated with the individual axes of the accelerometer, gyroscope, and magnetometer to improve accuracy for fall-direction classification. This section provides a detailed analysis of these equations.

(1) $$Acc_{M}=\sqrt{{Acc_{x}}^{2}+{Acc_{y}}^{2}+{Acc_{z}}^{2}}$$

(2) $$Gyr_{M}=\sqrt{{Gyr_{x}}^{2}+{Gyr_{y}}^{2}+{Gyr_{z}}^{2}}$$

(3) $$Mag_{M}=\sqrt{{Mag_{x}}^{2}+{Mag_{y}}^{2}+{Mag_{z}}^{2}}$$

In total, we had 12 channels (9 raw data + 3 magnitudes data) per wearable sensor: three magnitudes ($Acc_{M},\;Gyr_{M},\;\mathrm{and}\;Mag_{M}$), and the individual *x*, *y*, and *z* axes from the accelerometer, gyroscope, and magnetometer. Table 3 summarizes all raw data (*R*) and magnitudes (*M*) for feature extraction processing.

#### 2.3.2. Feature Extraction

Feature extraction is crucial for machine learning classification in fall-detection systems. We used time- and frequency-domain techniques, applying a sliding-window approach with 600 samples (10 s of data) per window. In the time domain, we extracted 8 features: maximum, minimum, standard deviation, sum of absolute values, root mean square (RMS), mean, range, and maximum difference between consecutive values. In the frequency domain, we extracted 10 features, including the maximum, minimum, standard deviation, sum of absolute values, RMS, kurtosis, skewness, mean, range, and the maximum difference between consecutive fast Fourier transform (FFT) values. Each IMU sensor produced 12 channels multiplied by 18 features from both domains, so the total is 216 values (data point) per sensor.

#### 2.3.3. Employing the Machine Learning Algorithm and Evaluation Method

We compared four commonly used supervised learning classification algorithms for fall-detection systems: RF [8], KNN [13], SVM [30], and MLP [10]. The optimal hyperparameters for each algorithm, identified through tuning, are listed in Table 4.

We evaluated our method using four key metrics: accuracy (4), precision (5), recall (sensitivity) (6), and F-score (7). Testing on a dataset partition showed exceptionally high accuracy and F-score. Testing on a dataset partition demonstrated exceptionally high accuracy and F-score, reflecting distinct patterns observed in each class—non-fall, backward fall, forward fall, and lateral fall. To ensure robustness, all results were derived using stratified 10-fold cross-validation to calculate the mean and standard deviation. This comprehensive analysis confirms that our system effectively distinguishes between different types of fall and non-fall activities, enhancing its overall performance.

(4) $$Accuracy=\frac{TP+TN}{TP+TN+FP+FN}$$

(5) $$Recall\;\left(sensitivity\right)=\frac{TP}{TP+FN}$$

(6) $$Precision=\frac{TP}{TP+FP}$$

(7) $$F-score=\frac{Recall\times Precision}{Recall+Precision}\times 2$$

In the evaluation formulas, TP represents True Positives, FN denotes False Negatives, FP stands for False Positives, and TN signifies True Negatives. 

We used the F-score (7) as a key metric for fall classification, offering a balanced and comprehensive evaluation of model performance [31]. This metric is particularly suitable for applications where both precision and recall are critical, especially in cases of class imbalance. We assessed performance using the mean of the F-score, validated statistically through the Kruskal–Wallis test to identify the most effective algorithm, and the t-test to check for significant differences in performance (f1_scores) between sensors on the left and right sides of the same area. We used the Kruskal–Wallis test to evaluate the statistical significance of performance differences across various sensor combinations. The *p*-values, being much larger than the typical significance threshold (e.g., 0.05), indicated no significant difference, supporting the null hypothesis. Post hoc testing with Tukey’s Honest Significant Difference (HSD) was conducted using estimated means (circles within bars) with 95% confidence intervals to identify the most effective sensor combinations for each body part.

## 3. Results

This section evaluates how sensor quantity and placement affect fall-direction classification accuracy using the most effective sensor combinations. Our objectives are to (i) identify optimal machine learning algorithms, (ii) analyze differences between left and right sensor placements, and (iii) determine the best sensor combinations for each body part to identify the most effective placements.

### 3.1. Analysis of the Effect of Machine Learning Algorithm Selection on Fall Classification Performance

Our aim is to select a single classifier based on its overall performance across the 12 sensors, ensuring simplicity and efficiency in determining the optimal classifier for fall-direction classification. We evaluated four popular supervised learning algorithms—RF, SVM, MLP, and KNN—to determine the highest-performance single classifier for fall-direction classification across different sensor locations. A Kruskal–Wallis test was conducted to examine statistically significant differences (*p* < 0.05) in performance between these classifiers across 12 sensor locations. The results indicated that SVM consistently outperformed the other classifiers, including MLP, with statistically significant differences in performance for several sensor locations.

While the Kruskal–Wallis test revealed significant differences between the classifiers, we conducted a pairwise Tukey HSD test to specifically compare the F-scores between SVM and MLP across all sensor locations. The Tukey HSD test showed that the difference in F-scores between SVM and MLP was statistically significant (*p* < 0.05) for key sensor positions, including the right shoulder and left upper leg, where SVM demonstrated significant improvement over MLP. For the remaining sensor positions, although no significant differences were found (*p* > 0.05), SVM still maintained higher average F-scores compared to MLP.

Additionally, the ranking of the classifiers based on their mean F-scores across all sensor locations consistently showed SVM as the top-performing classifier, with MLP following closely but not outperforming SVM significantly in most cases. These findings confirm that SVM is the most effective classifier for fall-direction classification across the 12 sensor locations, with statistically significant improvements over MLP for certain critical sensors. Table 5 summarizes the comparison of machine learning algorithms across the 12 sensor locations, highlighting the significant differences in performance between SVM and MLP and the overall ranking of the classifiers based on their mean F-scores. 

### 3.2. Results for Analyzing Sensor Location of Different Sides (Left and Right) on the Body

We compared the sensor locations on the left and right sides of the body to determine the most effective representative for each anatomical area. This analysis aimed to streamline sensor placement while maintaining data accuracy. After identifying the SVM algorithm as the best-performing algorithm (Section 3.1), we used it to compare corresponding sensor locations across five areas: shoulders, upper arms, forearms, upper legs, and lower legs.

Table 6 shows that the t-test analysis found no significant performance differences between left and right sensor locations within the same anatomical areas (shoulders, upper arms, forearms, upper legs, and lower legs). All *p*-values were greater than 0.05, indicating that sensors on both sides perform similarly. The t-test results for these comparisons are summarized in Table 6.

### 3.3. Results of Identifying Optimal Sensor Combinations and Comparing Body Parts for Effective Placement

We analyzed combinations of four upper-body sensors and three lower-body sensors for optimal fall-direction classification. After identifying SVM as the most effective model and finding no significant performance differences between sensors on the same anatomical area, we selected the best-performing sensors: head, right shoulder, right forearm, and left upper arm for the upper body; pelvis, left upper leg, and right lower leg for the lower body.

#### 3.3.1. Results of Identifying Four Different Sensor Location Combinations on the Upper Body Part

**Figure 2:**
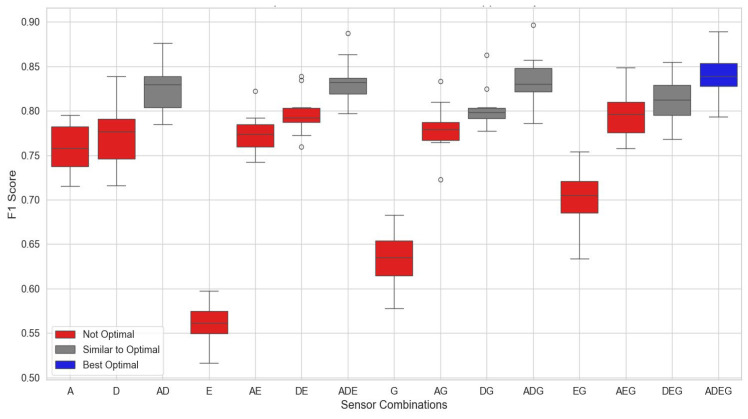
Results of identifying sensor combination on the upper body part, with positions indicated as follows: A: head, D: right shoulder, E: left forearm, and G: right upper arm.

We analyzed 17 sensor combinations on the upper body to identify the most effective locations, focusing on sensors positioned on the head, right shoulder, right forearm, and left upper arm. The Kruskal–Wallis test revealed a highly significant difference between the sensor combinations (*p* < 0.0001), indicating that sensor placement significantly affects the F1 score. A subsequent Tukey’s HSD test pinpointed the specific combinations driving these differences, with the best sensor locations illustrated in Figure 2.

Using these sensors, both individually and in combination, significantly improved algorithm performance compared with other configurations. As shown in Figure 2, six sensor setups—head–shoulder combination (AD), shoulder–upper arm (DG), shoulder–upper arm–forearm (DGE), head–shoulder–upper arm (ADG), head–shoulder–forearm (ADE), and all four sensors combined (ADEG, blue color)—achieved a mean F-score of approximately 0.82 and performance level, respectively. In contrast, sensors on the forearm and upper arm alone did not enhance system performance, indicating they may be less effective and could potentially reduce the overall accuracy of the fall-detection system.

**Figure 3:**
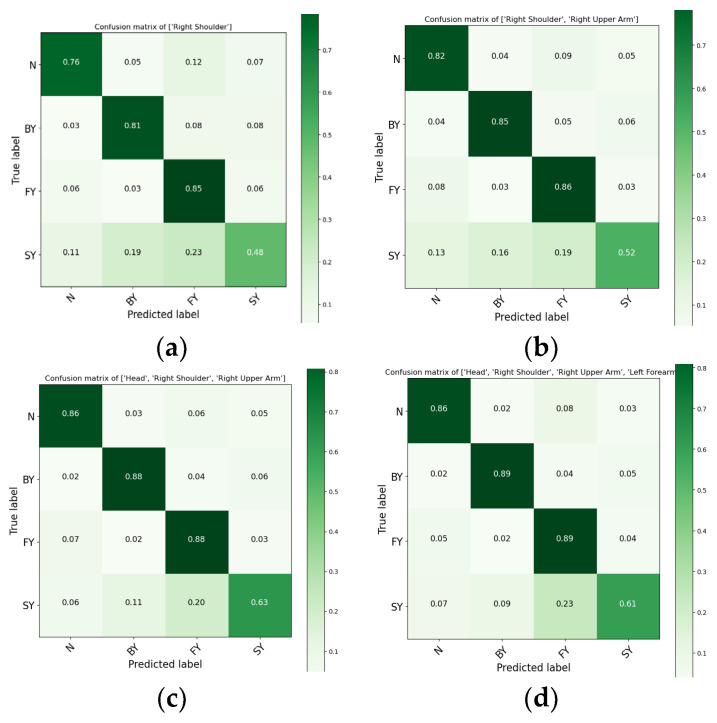
Confusion matrices for fall-direction classification based on optimal upper-body sensor locations: (**a**) shoulder-only sensor, (**b**) shoulder and upper arm combination, (**c**) head, shoulder, and upper arm combination, (**d**) head, shoulder, upper arm, and forearm combination. Classes are represented as “N” for non-fall, “BY” for backward fall, “FY” for forward fall, and “SY” for lateral fall.

The shoulder alone achieved a mean F-score of 77.40% (±3.68%), while the head achieved 75.74% (±2.86%). Performance improved when these positions were combined. For example, the shoulder–head combination yielded 82.64% (±2.83%), and the shoulder–upper arm combination reached 80.30% (±2.48%). Higher performances were observed when the three positions (shoulder, head, and upper arm) were combined, which resulted in a mean performance of 83.46% (±2.95%). The highest performance, 84.16% (±2.75%), was achieved when all four sensors were used together. These findings suggest that combining multiple sensor positions enhances the performance of fall classification systems. Figure 3 presents a confusion matrix based on the accuracy of each class, which evaluates the accuracy of various sensor combinations, helping to identify the most effective sensor placements for precise fall classification and making them more effective for real-world applications. While multiple sensor locations can enhance classification rates, focusing on key locations—such as the head, shoulder, and upper arm—proves most effective when used in combination. The confusion matrix (Figure 3) shows high precision and recall for non-fall and backward-fall events. However, slightly lower accuracy was observed for lateral falls, suggesting areas for improvement. Misclassifications primarily occurred between forward and lateral falls. Sensor combinations from the upper body, as described by the confusion matrix, are illustrated in Figure 3.

#### 3.3.2. Results of Three Different Sensor Location Combinations on the Lower Body

**Figure 4:**
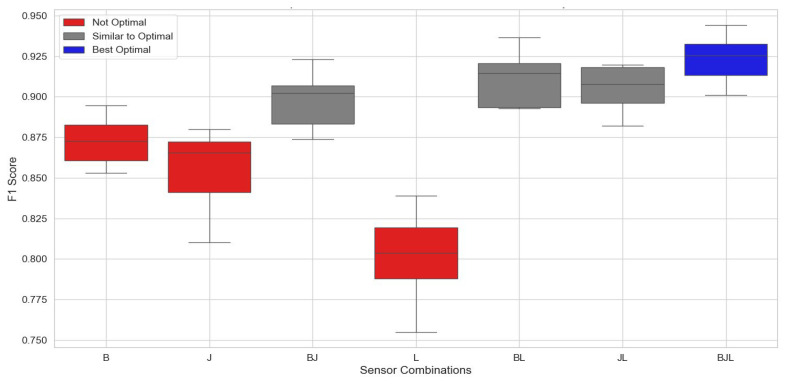
Results of identifying sensor combinations on the lower body, where positions are indicated as B: pelvis, J: left upper leg, and L: right lower leg.

**Figure 5:**
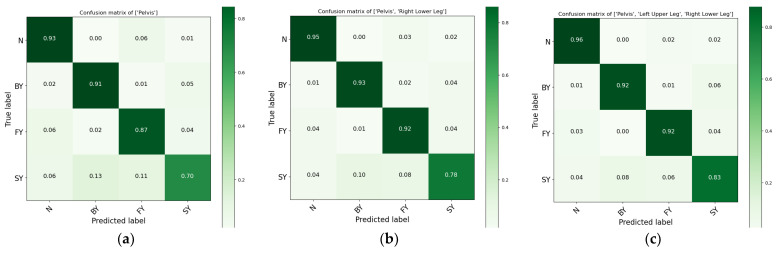
Confusion matrices for fall-direction classification based on optimal sensor locations on the lower body are presented as follows: (**a**) confusion matrix obtained from the pelvis location, (**b**) confusion matrix obtained from two combinations of pelvis–lower leg locations, (**c**) confusion matrix obtained from three combinations of pelvis–upper leg–lower leg locations. The classes are represented as “N” for “non-fall,” “BY” for “backward-fall,” “FY” for “forward-fall,” and “SY” for “lateral-fall” or “side-fall”.

In this analysis, we focused on identifying the optimal single-sensor and sensor combinations for the lower body using an evaluation method similar to that described in the previous section. The results demonstrated that the best performance for single sensors in the lower body was achieved with sensors placed on the pelvis (B) and upper legs (J), as illustrated in Figure 4. These sensors, when used individually, significantly enhanced the effectiveness of the algorithm, outperforming other lower-body sensors. In Figure 5, three configurations—the pelvis–lower leg (BL), upper leg–lower leg (JL), and all three sensors combined (BJL)—achieved a geometric mean of approximately 0.915. Sensors on the lower leg alone did not improve performance, but when combined with sensors in other locations, they enhanced overall performance. This figure presents Tukey’s HSD test results with a boxplot, comparing the mean F-score obtained using the SVM algorithm across these sensor positions. This analysis identifies the most effective sensor positions and combinations for accurate fall classification in the lower body parts, as shown in Figure 4.

For the lower body, the most effective areas were the pelvis at 87.24% (±1.38%) and the upper leg at 85.69% (±2.37%). Performance improved when these positions were combined. For example, combining the pelvis and lower legs resulted in a performance mean of 91.06% (±1.64%), while the combination of the upper and lower legs achieved 90.55% (±1.36%). The highest performance, 92.28% (±1.38%), was observed when all three locations—pelvis, upper leg, and lower leg—were combined. These findings indicate that combining multiple sensor positions generally enhances the performance of fall classification systems. Figure 5 presents the confusion matrix that highlights the performance of various sensor combinations for lower body parts. This analysis identifies the most efficient sensor locations and groupings for precise fall classification, enhancing their effectiveness for real-world applications. Combining multiple sensor locations, particularly the pelvis, upper leg, and lower leg, improves classification rates. The model performed exceptionally well in identifying non-fall, backward-fall, and forward-fall events with high precision and recall. However, lateral-fall classification showed slightly lower performance, indicating potential areas for model improvement. Misclassifications between forward and lateral falls were observed, likely due to similarities in fall patterns. The sensor combinations resulting from the lower body, as shown by the confusion matrices, are illustrated in Figure 5.

## 4. Discussion

Our results identified the shoulder and head as the most effective sensor locations for fall-direction classification on the upper body and the pelvis and upper leg for the lower body. These findings are consistent with previous studies that highlight the waist, chest, and thigh as key sensor locations (see Table 1) [5,7,15,16,31]. Although single-sensor placements are often sufficient for general fall detection, our study shows that they are inadequate for accurate fall-direction classification. Instead, combining sensors across both upper and lower body parts proved more effective. This approach enhances crucial data capture and simplifies the identification of the most effective sensor locations for multiclass fall classification and ADL systems.

We evaluated four classifiers across 12 sensor locations to select the best single classifier for fall-direction classification, with the SVM classifier emerging as the best classifier. This finding is consistent with previous studies that primarily focused on fall detection rather than fall-direction classification [7,16]. In those studies, SVM consistently outperformed other classifiers, including random forest (RF), multilayer perceptron (MLP), and K-Nearest Neighbors (KNN), in detecting falls from activities of daily living. Although some studies indicate that RF or MLP may perform better in specific fall-detection scenarios, our evaluation of fall-direction classification across various sensor locations demonstrated that SVM provided superior accuracy and consistency. Further research may explore the performance of alternative classifiers specifically for fall-direction classification.

We investigated whether sensor placement on the left versus right side of the body within the same anatomical area affected performance. Our analysis, covering five body areas (shoulders, upper arms, forearms, upper legs, and lower legs), found no significant performance differences between sides. This suggests that sensor placement within the same anatomical area can be flexible, allowing for random or alternating side selection without significantly affecting classification accuracy. This flexibility is beneficial for designing wearable systems as it can be adapted to user comfort or physical constraints.

While our results showed that combining sensors on multiple lower limb segments (pelvis, upper leg, and lower leg) provides the highest accuracy, we recognize that practical factors such as portability, affordability, and usability are just as important as accuracy in real-world applications of fall-detection systems. To maximize these practical aspects, we identified the pelvis as the single best sensor location for fall-direction classification. The pelvis offers a high classification performance due to its proximity to the center of mass, and it provides reliable data for detecting fall dynamics without requiring multiple sensors on the lower body. This makes it the most feasible choice for a single-sensor location, balancing accuracy with usability and cost-effectiveness. 

Comparing the lower body to the upper body, the combination of the pelvis, upper leg, and lower leg sensors outperformed upper-body sensor combinations in classifying fall directions. The highest accuracy of 92.28% (±1.38%) was achieved with the three lower-body sensors, while upper-body sensors, such as the combination of shoulder, head, and upper arm, achieved a maximum accuracy of 83.46% (±2.95%). This suggests that lower-body sensors provide more accurate data for classifying fall direction, likely due to their closer proximity to the center of mass and the points of impact during a fall. These findings indicate that lower-body sensors should be prioritized in future wearable fall classification systems, as they significantly enhance classification accuracy for fall events. 

However, we recognize the possibility that combining sensors from both upper and lower body regions could provide a more comprehensive view of body movement during a fall. This combination may improve the overall classification accuracy by leveraging complementary data from both areas. Therefore, future work could explore optimal combinations of upper and lower body sensors to maximize accuracy while minimizing the number of sensors required.

For the misclassifications between forward and lateral falls, contrary to initial assumptions about similarities in fall patterns, previous studies suggest that forward and lateral falls exhibit distinct biomechanical characteristics [32]. Forward falls typically involve different body segments and impact forces compared to lateral falls. Therefore, this first reason, the observed misclassifications may stem from sensor placement limitations or challenges in detecting lateral falls using the current setup. Lower-body sensor placements (pelvis, upper leg, and lower leg) also demonstrated superior classification performance compared to upper-body placements. This may indicate that lower-body sensors capture fall dynamics more effectively, especially in lateral falls, where the legs and pelvis may experience more pronounced movement and impact. And the second reason is the misclassification by a similar pattern. Further investigation into optimizing sensor configurations or applying more advanced machine learning models could help improve the detection of lateral falls.

Another observation is that lateral falls (SY) were misclassified as backward falls (BY) more frequently than as forward falls (FY), as shown in Figure 5. This is due to the similarities in body movement during backward and lateral falls, especially in the lower body. Both backward and lateral falls involve shifting of weight away from the center of mass, causing the legs and pelvis to experience similar patterns of acceleration and deceleration, particularly when using the current sensor placements. Forward falls, on the other hand, typically involve more pronounced upper body movement, which may make them easier to differentiate from lateral falls. The higher misclassification rate for lateral falls (SY) compared to other fall directions may also be attributed to the complex nature of lateral falls, where sideways movement of the body, particularly the legs and pelvis, produces sensor data that are more challenging to distinguish from other fall types, especially backward falls. This could indicate the need for further optimization of sensor placement or the addition of sensors specifically tailored to capture the unique dynamics of lateral falls.

Our results showed that lower-body sensor placements are more effective than upper-body sensor placements for fall-direction classification. The optimal combination involves three locations on the lower body: pelvis, upper leg, and lower leg, that significantly improved classification accuracy. Therefore, we recommend prioritizing the lower body for configuring sensor placements to collect essential data for accurate fall-direction classification.

This study has limitations. It focused on 12 specific sensor locations, excluding other potentially valuable locations such as the chest and neck. Although the SVM was the most effective model for this dataset, other machine learning or deep learning models not explored here may yield different results. The dataset may not represent the full range of daily activities, potentially limiting the robustness of the system in real-world scenarios. Lastly, although no significant differences were found between left and right sensor placements, this may not apply universally, as individual variations were not fully explored.

Additionally, the study population was not specifically at high risk for falls. The participants, while capable of imitating fall behaviors, were not elderly or mobility-impaired individuals, which may affect the applicability of our results to populations with a higher risk of falling. Falls in real-life scenarios are often unintentional and accidental, and these events may exhibit different biomechanical characteristics compared to the self-initiated falls simulated in our study. Real-life falls may involve higher levels of unpredictability, acceleration, and dynamic movement, which could influence the sensor data, particularly acceleration and gyroscope values. Therefore, while the falls in this study were designed to mimic high-risk behaviors, we acknowledge that self-initiated falls may not fully capture the complexities of accidental falls, limiting the generalizability of our findings. Future research should explore fall detection and classification in populations at higher risk and in more naturalistic, real-world settings to better understand the optimal sensor configurations for real-life fall scenarios. 

One key limitation is that we did not account for changes in fall direction during the event. In real-life scenarios, a person may begin falling in one direction, such as forward, but rotate mid-fall and land on their side [27]. This dynamic shift in fall direction was not considered in our study, as our simulated falls followed a single, consistent trajectory. Future research should explore this aspect to better replicate real-world falls and improve the robustness of fall-direction classification systems.

## 5. Conclusions

This study aimed to optimize sensor placement on the human body for fall-direction classification while minimizing the number of sensors used. We focused on three main objectives: identifying the most effective machine learning classifier, analyzing whether sensor placement on different sides of the body impacts performance, and determining the optimal combination of upper- and lower-body sensors for accurate fall-direction classification.

Our findings highlight that while multiple sensors generally improve classification accuracy, focusing on two or three key sensor locations can provide robust performance. Specifically, the pelvis, upper leg, and lower leg emerged as the most effective lower-body locations, while the head, shoulder, and upper arm were identified as the best-performing upper-body locations. Although a single sensor can reliably distinguish between fall and non-fall events, it is less effective for identifying specific fall directions. Combining two or three sensors significantly enhances directional classification, achieving accuracies of 96% for non-fall events, 92% for backward and forward falls, and 83% for lateral falls. This demonstrates the importance of sensor placement, especially in areas like the pelvis and lower limbs, which are closer to the center of mass and impact points during a fall.

Future research should explore additional sensor placements beyond the 12 anatomical locations analyzed in this study to further improve fall-direction classification. Additionally, the application of advanced machine learning techniques, including deep learning models, could provide enhanced accuracy by capturing more complex movement patterns. Expanding the dataset to include a wider range of daily activities and fall types will be critical for improving the real-world applicability of fall-detection systems. Furthermore, individual variations—such as body type, age, and fall risk—should be explored to develop more personalized and effective fall classification solutions.

These future directions are essential for advancing the field of fall-direction classification, ensuring that the systems developed are not only highly accurate but also practical and adaptable, ensuring their effectiveness in diverse real-world scenarios, especially for populations at higher risk of falls.

## Figures and Tables

**Table 2:** The number of simples for each class and the maximum row count per sample.

Fall Type	Simple Each Class	Maximum of Row Each Simple
None fall	720	600
Backward	736	600
Forward	977	600
Side	438	600
Total	2871	600

**Table 3:** Description of all raw data and magnitudes as input for feature extraction processing.

No,	Raw Data	Description
*R_1_*–*R_3_*	*Acc* (*x*, *y*, *z*)	3-axis of an accelerometer
*M_1_*	$Acc_{M}$	1-scaler value from a 3-axis of an accelerometer
*R_4_*–*R_6_*	*Gyr* (*x*, *y*, *z*)	3-axis of a gyroscope
*M_2_*	$Gyr_{M}$	1-scaler value from a 3-axis of a gyroscope
*R_7_*–*R_9_*	*Mag* (*x*, *y*, *z*)	3-axis of a magnetometer
*M_3_*	$Mag_{M}$	1-scaler value from a 3-axis of a magnetometer

**Table 4:** Supervised machine learning models and parameters.

Models	Structure	Best Parameters
RF	Ensemble of decision trees	n_estimators=100, bootstrap=False, max_features=s‘qrt’.
SVM	Nonlinear classification model	kernel=r‘bf’, C=10, gamma=s‘cale’.
MLP	Feedforward neural network	hidden_layer_sizes=(100,), max_iter=1000, activation=r‘elu’, alpha=0.0001, solver=s‘gd’.
KNN	Instance-based learning	n_neighbors=10, metric=m‘anhattan’, weights=u‘niform’.

**Table 5:** Results from comparing machine learning algorithms.

Sensor		A	B	C	D	E	F	G	H	I	J	K	L
**Classifiers**	SVM	75.74	87.24	76.53	77.40	56.14	57.16	66.37	63.49	85.69	84.30	79.01	80.21
	MLP	73.48	85.33	75.04	73.91	55.09	54.92	65.23	61.10	83.76	82.70	76.57	78.75
	*p*-value	0.112	0.082	0.364	0.023 *	0.364	0.326	0.406	0.131	0.096	0.049 *	0.082	0.199
	RF	66.93	82.02	70.85	69.66	51.68	52.63	59.13	58.78	78.69	78.55	74.50	75.93
	KNN	66.52	79.26	68.72	68.87	46.78	48.57	56.41	54.71	77.06	77.90	70.25	71.28
	*p*-value	0.705	0.028 *	0.174	0.597	0.008 **	0.003 **	0.174	0.082	0.257	0.290	0.000 ***	0.001 ***

Note: * *p* < 0.05, ** *p* < 0.01, *** *p* < 0.001, *p*-values below 0.05 indicate statistically significant differences in classifier performance across the respective sensor locations. The sensor locations are designated as follows: A (head), B (pelvis), C (left shoulder), D (right shoulder), E (left forearm), F (right forearm), G (right upper arm), H (left upper arm), I (right upper leg), J (left upper leg), K (left lower leg), and L (right lower leg).

**Table 6:** Statistical analysis results from comparing the left and right sides of the body.

Sensor Areas	Shoulders	Upper Arms	Forearms	Upper Legs	Lower Legs
T-statistic	−0.6534	−1.8727	−0.8059	−1.3912	−1.0864
*p*-value	0.5217	0.0774	0.4308	0.1811	0.2917

## Data Availability

The study’s data are available upon request from the corresponding author.
